# Surgery alone, adjuvant tegafur/gimeracil/octeracil (S-1), or platinum-based chemotherapies for resectable gastric cancer: real-world experience and a propensity score matching analysis

**DOI:** 10.1186/s12885-021-08487-z

**Published:** 2021-07-09

**Authors:** Chih-Chieh Yen, Yan-Shen Shan, Ying-Jui Chao, Ting-Kai Liao, I-Shu Chen, Hsuan-Yi Huang, I-Ting Liu, Chia-Jui Yen

**Affiliations:** 1 Division of Hematology/ Oncology, Department of Internal Medicine, National Cheng Kung University Hospital Douliou Branch, Yunlin, Taiwan; 2 Institute of Clinical Medicine, School of Medicine, National Cheng Kung University, Tainan, Taiwan; 3 Department of Surgery, National Cheng Kung University Hospital, College of Medicine, National Cheng Kung University, Tainan, Taiwan; 4 Department of Surgery, National Cheng Kung University Hospital Douliou Branch, YunLin, Taiwan; 5 Department of Surgery, Kaohsiung Veterans General Hospital, Kaohsiung, Taiwan; 6 Division of Colorectal Surgery, Department of Surgery, Chi Mei Medical Center, Tainan, Taiwan; 7 Department of Oncology, National Cheng Kung University Hospital, College of Medicine, National Cheng Kung University, No. 138, Sheng-Li Road, Tainan, 70403 Taiwan

**Keywords:** Adjuvant, Resectable gastric cancer, S-1, Platinum, Observation

## Abstract

**Background:**

Adjuvant chemotherapy has changed the paradigm in resectable gastric cancer. S-1 is an oral chemotherapeutic with promising efficacy in Asia. However, comparisons with close observation or platinum-based doublets post D2 gastrectomy have been less reported, notably on real-world experiences.

**Methods:**

We retrospectively evaluated patients with D2-dissected stage IB-III gastric cancer who received S-1 (S-1, *n* = 67), platinum-based doublets (P, *n* = 145) and surgery with close observation (OBS, *n* = 221) from Jan 2008 to Oct 2018. A propensity score matching was used to compare for recurrence-free (RFS) and overall survivals (OS) in patients who had a locally-advanced disease (T3–4 or lymph node-positive). Adverse reactions, dosage, and associated factors for S-1 are also discussed.

**Results:**

In a median follow-up time of 51.9 months, adjuvant S-1 monotherapy was associated with an intermediate survival as compared with P and OBS (median RFS/OS: S-1 vs. P, 20.9/35.8 vs. 31.2/50.5 months, HR = 1.76/2.14, *p* = 0.021/0.008; S-1 vs. OBS, 24.4/40.2 vs. 20.7/27.0 months, HR = 0.62/0.55, *p* = 0.041/0.024). The survival differences were more prominent in patients with N2–3 diseases. S-1 was well-tolerated with a relative dose intensity of 73.6%, a median duration of 8.3 months and associated with less adverse reactions as compared with P. S-1 monotherapy was selected by physicians based on age, lymph node stage, serum carcinoembryonic antigen and disease stage.

**Conclusions:**

Adjuvant S-1 correlated with intermediate survival outcomes between OBS and P but conferred fewer adverse reactions as compared with P. Patients with a moderate risk of recurrence had comparable survivals when treated with S-1 while platinum-based doublets were favored in advanced cases. The study provides additional information about adjuvant S-1 in patients with selected risk of recurrence.

**Supplementary Information:**

The online version contains supplementary material available at 10.1186/s12885-021-08487-z.

## Background

Among all primary gastrointestinal malignancies globally, gastric cancer is highly prevalent and ranks as the 3rd most deadly cancer [1]. The incidence of gastric cancer varies with distinctive geographic and ethnic distributions, where countries in Eastern/Central Asia, Latin America, and Eastern Europe are commonly affected [2]. Despite emerging improvements in early detection and curative treatment, nearly two thirds of high-risk patients still encounter a recurrence or progression of the disease [3]. Therefore, adjuvant or perioperative chemotherapies have been intensively investigated in recent years to reduce distant metastasis [4–6]. Interestingly, the Eastern and Western world share different perspectives of the same disease, in that the former tends to have patients diagnosed earlier, have more lymph nodes dissected, have biologically favorable tumors, and receive more adjuvant chemotherapies and fewer concurrent chemoradiotherapies (CCRT) [7]. However, given aggressive salvage therapies, patients with recurrent or advanced gastric cancer still have a dismal outcome with survival barely exceeding more than one year [8].

S-1, or TS-1, is an orally available chemotherapeutic composed of tegafur (a prodrug of fluorouracil, 5-FU), gimeracil (preventing dihydropyrimidine dehydrogenase-mediated degradation of 5-FU), and oteracil (reducing the toxic effects of 5-FU) [9]. It is well-tolerated without serious adverse reactions, easily implemented in an outpatient setting, and currently approved for gastric, lung, breast, and biliary tract cancers in several European and Asian countries but not in the United States [10]. S-1 is a key drug replacing the 5-FU backbone and has been incorporated with various chemotherapeutic partners in the treatment of advanced or metastatic gastric cancer in Japanese patients [11–13]. In addition, S-1-based adjuvant therapies, either as mono- or combinatorial regimens, have been confirmed to have efficacy in patients with gastric cancer who have received curative gastrectomy and adequate lymph node dissection (D2 or above) in phase III studies [14, 15]. Ongoing studies are investigating neoadjuvant or perioperative chemotherapies containing S-1 as promising options for patients with borderline resectable, locally-advanced, or high-risk gastric cancer [16].

Although S-1 has extended the therapeutic options in adjuvant therapy for gastric cancer, most of the published studies have incorporated patients from Northeast Asia. The extrapolation of the results to other Asian or non-Asian countries remains undetermined. Conversely, genetic and ethnic polymorphisms contribute to differences in drug metabolism, interactions, and dose-limiting toxicities. Pharmacokinetic studies revealed a significantly lower tolerable dose of S-1 in Caucasian patients [17]. In addition, real-world experiences outside of clinical trial settings have been less reported, in which the efficacy of S-1 is potentially confounded by compliance, availability, and undesirable adverse events. In the absence of a universal consensus related to the effective use of adjuvant chemotherapies for resectable gastric cancer, conventional regimens vary across countries. In general, platinum-based doublets, such as cisplatin or oxaliplatin plus a fluoropyrimidine, are widely recommended in the international guidelines [18]. Therefore, a comparison of the efficacy and tolerability of S-1 monotherapy with other adjuvant chemotherapies for patients with resectable gastric cancer warrants further studies, notably real-world experiences in daily clinical practice.

In the present study, we retrospectively evaluated patients with resectable gastric cancer who had received adjuvant S-1 monotherapy post D2 gastrectomy. Patients who received surgery alone or adjuvant platinum-based doublet chemotherapies were enrolled for the purpose of detailed comparisons. Characteristics and survival outcomes were assessed. Also, we reviewed the adverse events and associated factors for S-1 other than platinum-based regimens as selected by physicians. Owing to the differences in baseline disease severity and the nature of retrospective observations, propensity score matching was conducted to enhance between-group comparability. A landscape of recent S-1-based adjuvant or perioperative therapies for gastric cancer was reviewed as well.

## Materials and methods

### Patients

We included 433 eligible Han Chinese patients with resectable gastric (cardia, fundus, body, antrum, and pylorus) or gastroesophageal junction (GEJ) (Siewert type III tumor) cancer from Jan 2008 to Oct 2018. All patients had an initial American Joint Committee on Cancer (AJCC) pathological staging, 8th edition of IB to III or a re-staging of IB to III disease post neoadjuvant therapies. Patients were treated and evaluated at National Cheng Kung University Hospital and its affiliated branches in Taiwan, with an average volume of radical gastrectomy of 90 cases per year. Eligible patients were required to have received radical gastrectomy (laparotomic, laparoscopic or robotic) plus perigastric and celiac axial lymph node dissection (D2) in R0 resection without residual tumors. Adjuvant chemotherapies were defined as oral or intravenous administration of any single or combinatorial chemotherapeutics within 12 weeks post-operatively for at least two cycles or for 6 consecutive weeks. Eligible chemotherapeutics included S-1 (TTY Biopharm, Taiwan), 5-FU, leucovorin, cisplatin, oxaliplatin, and capecitabine, which were selected based on the physician’s clinical judgment. Patients were allocated according to the chosen adjuvant chemotherapies as S-1 (Group S-1) or platinum-based doublets (Group P). Those who received close observation with active surveillance were deemed as surgery alone (observation, Group OBS). Patients were excluded if they had carcinoma in situ, initial metastatic or recurrent diseases, adenocarcinoma of unspecified primary sites, secondary cancer with gastric invasion, gastric or GEJ squamous cell carcinoma, small cell carcinoma, lymphoma, or neuroendocrine carcinoma.

### Clinical evaluations

Clinical and pathological features, including demographic characteristics, biochemical markers, *Helicobacter pylori* (*H. pylori*) infection, human epidermal growth factor receptor-2 (HER2) status, and treatment outcomes were evaluated based on written or electronic medical records. Chemotherapy-related adverse events were retrospectively retrieved from documented materials and graded according to the Common Toxicity Criteria of the National Cancer Institute (CTCAE) version 4.0. Routine or on-demand esophagogastroduodenoscopy, imaging studies (computed tomography or magnetic resonance imaging), and biopsied or cytological examinations were arranged according to the local practice guidelines and incorporated as the detection of disease recurrence or progression. Recurrence-free survival (RFS) was defined by the date of diagnosis to first documented recurrence or progression of the disease, physician-initiated subsequent therapy, or the death of the patient due to any cause. Overall survival (OS) was defined by the date of diagnosis to the death of the patient due to any cause.

### Statistical analysis

We presented the clinical and pathological features in descriptive analyses as percentages. Continuous variables were compared using a Student’s t-test or a one-way analysis of variance (ANOVA) plus a post-hoc Tuckey honestly significant difference (Tuckey’s HSD) test, whereas categorical variables were compared using a Chi-squared test or Fisher’s exact test.

The variables that did not meet the parametric assumptions were evaluated using non-parametric methods. To enhance the robustness of comparability within the study groups, we first selected patients with a locally-advanced disease (T3-T4, lymph node (LN)-involved (N1 at least), or both) indicated for adjuvant therapies [19, 20]. Stage IB LN-negative (pT2N0M0) patients were excluded. Propensity scores (PPS) were calculated using a multivariate logistic regression adjusted for relevant covariates. We then matched patients by age, performance status, AJCC stage, tumor (T) stage, lymph node (N) stage and serum carcinoembryonic antigen (CEA) level to balance the potential heterogeneity and tumor burden. We conducted PPS matching using the nearest neighbor method at a ratio of 1:2 with a caliper of 0.25 to reduce selection bias. If the standardized mean differences are less than 10%, the covariates are considered balanced between the two groups. In addition, we calculated RFS and OS using a Kaplan-Meier survival estimation and compared it with log-rank test. Uni- and multivariate binary logistic regression analyses were conducted to elucidate independent associated factors for selecting S-1 as adjuvant chemotherapy. Statistical significance was prespecified by *p* < 0.05. We used GraphPad Prism 7.0® (GraphPad software, CA, US), SAS 9.4® (SAS Institute Inc., NC, US) and R® 3.5.1 for data management and graphics.

## Results

### Patient enrollment and baseline characteristics

**Fig. 1:**
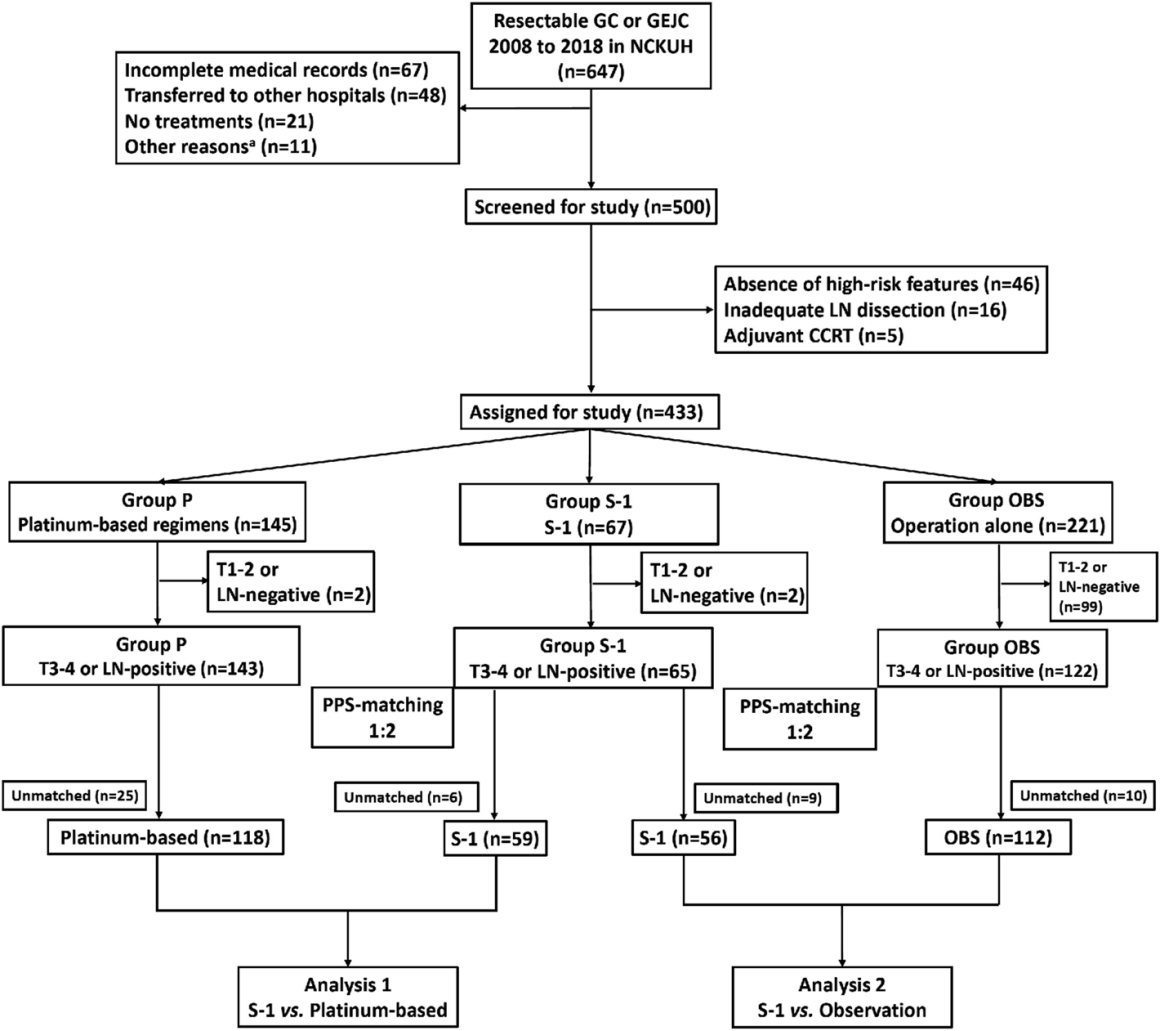
Flow diagram of the study. ^a^Concomitant malignancies other than gastric cancer (n=6), metastatic carcinoma not confined to gastric origin (*n* = 3), and esophageal carcinoma with gastric invasion (*n* = 2). GC, gastric cancer; GEJC, gastroesophageal junction cancer; NCKUH, National Cheng Kung University Hospital; CCRT, concurrent chemoradiotherapy; LN, lymph node; PPS, propensity score

The flow diagram of the study is shown in Fig. 1. A total of 647 patients were initially screened for enrollment from Jan 2008 to Oct 2018. After assessing the eligibility, 433 patients were included in the study. The patient and tumor characteristics are shown in Table 1. Among the included patients, 221 received curative gastrectomy without adjuvant therapies (Group OBS), 67 had S-1 (Group S-1), and 145 had platinum-based doublet chemotherapies (Group P). The distributions of gender, *H. pylori* infection, HER2 status, baseline performance status, GEJ tumors, and serum cancer antigen 19–9 (CA19–9) levels were similar among the three groups. The median ages were significantly elder in Group OBS, followed by S-1, and P, respectively (OBS vs. S-1 vs. P, 71.9 vs. 65.6 vs. 58.5 years). More patients had early stages of the disease, notably stage IB-II, T1–2 or N0 disease, in the Group OBS as compared with S-1 or P (OBS: stage IB-II, 72.4%; T1–2 59.3%; N0–1, 78.7%; all *p* < 0.05 when compared with S-1 or P). Similarly, Group S-1 had more stage IB-II and N0–1 patients as compared with P (S-1 vs. P: stage IB-II, 38.8% vs. 20.0%, *p* = 1.1e-6; N0–1, 44.8% vs. 26.9%, *p* = 0.006). Lymphovascular invasion (LVI) was more evident in Group S-1 and P as compared with OBS (OBS vs. S-1 vs. P: 38.9% vs. 70.1% vs. 80.7%). The median serum CEA was higher in Group P as compared with S-1 or OBS (OBS vs. S-1 vs. P: 2.2 vs. 1.8 vs. 2.6 ng/mL; *p* = 0.001 via a Kruskal-Wallis test). A total of 5 patients had received neoadjuvant therapies prior to surgery, where 2 had triplet and 3 had doublet chemotherapies.

**Table 1:** Patient characteristics

Group	OBS		S-1		P				
	**Operation alone** **(n = 221)**		**S-1** **(n = 67)**		**Platinum-based doublets** **(n = 145)**		*p*		
							OBS vs. S-1	OBS vs. P	S-1 vs. P
**Age, median (IQR)**	71.9	(62.6–79.3)	65.6	(56.1–76.0)	58.5	(50.0–66.6)	9.4e-3*	1.2e-6*	6.2e-4*
**Male, n (%)**	63	(57.3)	32	(50.0)	58	(61.1)	0.353	0.264	0.224
**AJCC stage, n (%)**							1.3e-4*	1.1e-6*	1.2e-4*
I-II	160	(72.4)	26	(38.8)	29	(20.0)			
III	61		41		117				
**pT stage, n (%)**									
T1/2	131	(59.3)	13	(19.4)	29	(20.0)	2.4e-8*	1.0e-6*	0.998
T3	64		33		58				
T4	26		21		58				
**pN stage, n (%)**							2.0e-7*	1.0e-8*	0.006*
N0–1	174	(78.7)	30	(44.8)	39	(26.9)			
N2–3	47		37		116				
**ECOG ≥ 2, n (%)**	23	(10.4)	5	(7.8)	11	(7.6)	0.477	0.468	0.804
**HER2-positive, n (%)**	15	(6.8)	4	(6.0)	10	(6.9)	0.964	0.863	0.964
**HER2-therapy**^**a**^**, n**	1		0		2				
**Tumor at GEJ, n (%)**	11	(5.0)	5	(7.5)	11	(7.6)	0.634	0.422	0.804
**Lauren histology, n (%)**							0.536	0.012*	0.291
Intestinal	94	(42.5)	25	(37.3)	42	(29.0)			
Diffuse	91	(41.1)	28	(41.8)	80	(55.2)			
Mixed	33		13		23				
Others^b^	3		1		0				
**Borrmann classification, n (%)**							1.3e-4*	1.0e-8*	0.283
Infiltrative	104	(49.1)	50	(74.6)	115	(79.3)			
Non-infiltrative	117		17		25				
**LVI (+), n (%)**	86	(38.9)	47	(70.1)	117	(80.7)	1.3e-5*	1.0e-8*	0.126
***H. pylori*** **infection, n (%)**	48	(21.7)	13	(19.4)	40	(27.6)	0.814	0.246	0.268
**Median CEA, ng/mL (IQR)**	2.2	(1.3–3.5)	1.8	(1.3–2.6)	2.6	(1.4–4.6)	0.143	0.027*	0.002*
**Median CA19–9, U/mL (IQR)**	13.2	(6.1–27.0)	13.7	(6.3–23.5)	14.1	(6.5–28.9)	0.555		
**Neoadjuvant therapies**^**c**^**, n (%)**	0		2	(3.0)	3	(2.1)	–	–	0.943
**Adjuvant chemotherapy, n**									
S-1	–		67		–				
PFL	–		–		81				
XELOX/FOLFOX	–		–		61				
Others^d^	–		–		3		–	–	–

a. Defined as any frontline or subsequent HER2-directed therapies in patients with HER2-positive disease. Group OBS, XELOX/trastuzumab (n = 1); Group P, XELOX/trastuzumab (n = 1) and lapatinib (n = 1)

b. Group OBS, lymphoepithelioma (*n* = 3); Group S-1, adenocarcinoma with neuroendocrine differentiation (n = 1)

c. Group S-1, DCF (n = 1) and FLOT (n = 1); Group P, XELOX (n = 3)

d. ECF (n = 3)

IQR, interquartile range; ECOG, Eastern Cooperative Oncology Group; GEJ, gastroesophageal junction; LVI, lymphovascular invasion; CCRT, concurrent chemoradiotherapy; PFL, cisplatin/fluorouracil/leucovorin; XELOX, capecitabine/oxaliplatin; FOLFOX, oxaliplatin/fluorouracil/leucovorin; DCF, docetaxel/cisplatin/fluorouracil; FLOT, docetaxel/oxaliplatin/fluorouracil/leucovorin; ECF, epirubicin/cisplatin/ fluorouracil

### PPS matching and post-match characteristics

Two independent 1:2 matched results are demonstrated in Table 2. A total of 59 versus 118 patients and 56 versus 112 patients were matched in the analysis 1 (S-1 vs. P) and 2 (S-1 vs. OBS), respectively. Both post-match analyses revealed well-balanced characteristics, with the exception of marginal disproportions in pN stage. In Group S-1, all patients had S-1 monotherapy as adjuvant therapy. In Group P, 60.2% of the patients had cisplatin/5-FU/leucovorin (PFL) and others had oxaliplatin-based doublets as the selected adjuvant therapies. Subsequent systemic therapies of the study population were shown in Supplementary 1.

**Table 2:** Post matching characteristics

	Analysis 1 (1:2)					Analysis 2 (1:2)				
Group	S-1		P-based			S-1		OBS		
	**S-1** **(*n*** **= 59)**		**Platinum-based (*n*** **= 118)**		*p*	**S-1** **(*n*** **= 56)**		**Surgery alone (*n*** **= 112)**		*p*
**Age, median (IQR)**	65.6	(57.3–76.7)	60.1	(51.1–68.2)	0.119	65.5	(56.0–74.7)	71.1	(61.2–75.5)	0.103
**Male, n (%)**	30	(50.8)	65	(55.1)	0.709	32	(57.1)	62	(55.3)	0.956
**Disease stage, n (%)**					0.671					0.252
I-II	17	(28.8)	29	(24.6)		24	(42.9)	60	(53.5)	
III	42		89			32		52		
**pT stage, n (%)**					0.442					0.397
T1–2	8	(13.6)	23	(19.5)		10	(17.9)	28	(25.0)	
T3–4	51		95			46		84		
**pN stage, n (%)**					0.288					0.208
N0–1	24	(40.7)	37	(31.3)		26	(46.4)	65	(58.0)	
N2–3	35		81			30		47		
**ECOG ≥ 2, n (%)**	4	(6.8)	9	(7.6)	0.919	4	(7.1)	12	(10.7)	0.642
**HER2-positive, n (%)**	3	(5.1)	8	(6.8)	0.945	4	(7.1)	10	(8.9)	0.921
**Tumor at GEJ, n (%)**	4	(6.8)	5	(4.2)	0.717	4	(7.1)	5	(4.5)	0.716
**Lauren histology type, n (%)**					0.232					0.781
Intestinal	23	(39.0)	34	(28.8)		21	(37.5)	46	(41.1)	
Diffuse	24	(40.7)	67	(56.8)		23	(41.1)	48	(42.9)	
Mixed	11		17			11		15		
Others^a^	1		0			1		3		
**Borrmann classification, n (%)**					0.945					0.676
Infiltrative	47	(60.7)	95	(70.5)		41	(73.2)	77	(68.8)	
Non-infiltrative	12		23			15		35		
**LVI (+), n (%)**	43	(72.9)	96	(81.4)	0.271	39	(69.6)	72	(64.3)	0.604
**Median CEA ng/mL, (IQR)**	1.8	(1.3–2.8)	2.4	(1.3–3.5)	0.213	1.9	(1.3–2.6)	2.1	(1.5–4.4)	0.072
***H. pylori*** **infection, n (%)**	9	(15.3)	34	(28.8)	0.072	10	(17.9)	23	(20.5)	0.837
**Adjuvant chemotherapy, n (%)**										
S-1	59		–			56		–		
PFL	–		71			–		–		
XELOX/FOLFOX	–		47			–		–		

a. Analysis 1: adenocarcinoma with neuroendocrine differentiation (n = 1); analysis 2: group S-1, adenocarcinoma with neuroendocrine differentiation (n = 1) and group OBS, lymphoepithelioma (n = 3)

### Survival: S-1 vs. platinum-based doublets

**Fig. 2:**
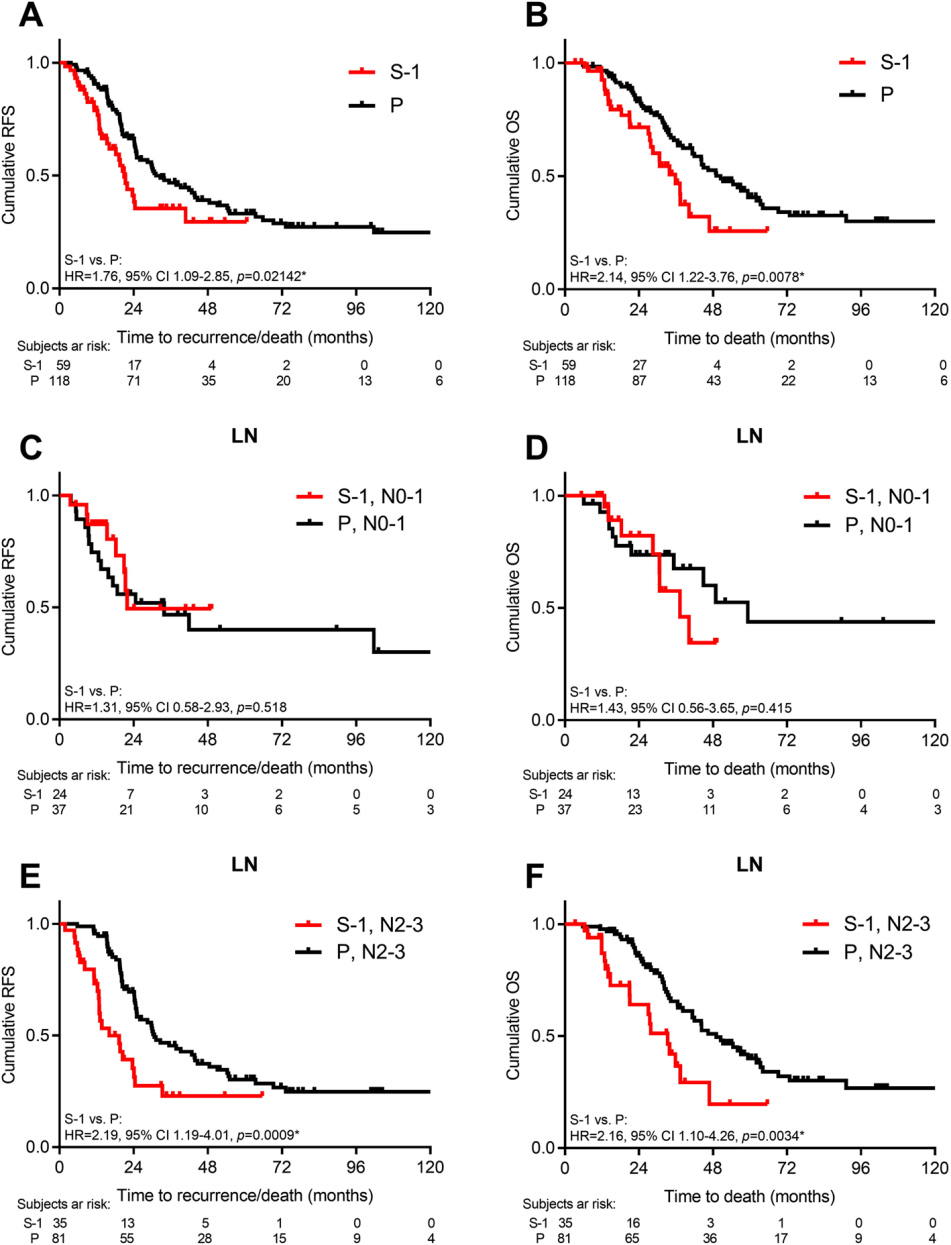
Survival: S-1 vs. platinum-based doublets. RFS and OS by adjuvant chemotherapy (**A**-**B**), lymph node stage of N2-3 (**C**-**D**), and N0-1 (**E**-**F**). Log-rank test, *p*< 0.05 as statistically significant and shown as *

All patients were evaluated in a median follow-up time of 51.9 months. In analysis 1, patients who received adjuvant S-1 had a significantly shorter RFS and OS as compared with platinum-based doublets (median RFS/OS: S-1 vs. P, 20.9/35.8 vs. 31.2/50.5 months, HR = 1.76/2.14, *p* = 0.021/0.008; Fig. 2A and B). Stratified by the N stage, the survival differences were not evident in terms of N0–1 disease (median RFS/OS: S-1 vs. P, 21.9/37.3 vs. 33.8/59.3 months, HR = 1.31/1.43, *p* = 0.518/0.415; Fig. 2C and D). However, Group P had a significantly better RFS and OS in N2–3 disease as compared with S-1 (median RFS/OS: S-1 vs. P, 16.1/33.3 vs. 30.7/49.1 months, HR = 2.19/2.16, *p* = 0.001/0.003; Fig. 2E and F). Similarly, patients with stage IB to II disease had comparable RFS and OS (median RFS/OS: S-1 vs. P, unreached/33.3 vs. unreached/51.2 months, HR = 0.43/0.61, *p* = 0.107/0.433; Supplementary 2A and 2B). However, group P was associated with significantly favorable survivals in patients with stage III disease (median RFS/OS: S-1 vs. P, 13.7/27.5 vs. 30.7/49.1 months, HR = 2.56/2.54, *p* = 1.9e-5/8.4e-5; Supplementary 2C and 2D). We did not observe significant survival differences based on S-1, oxaliplatin, or cisplatin-based chemotherapies in the matched population (median RFS: S-1 vs. oxaliplatin vs. cisplatin, 21.9 vs. 24.9 vs. 42.8 months; all *p* ≥ 0.05 between groups; Supplementary 2E) (median OS: S-1 vs. oxaliplatin vs. cisplatin, 35.8 vs. 44.3 vs. 57.0 months; all *p* ≥ 0.05 between groups; Supplementary 2F).

### Survival: S-1 vs. OBS

**Fig. 3:**
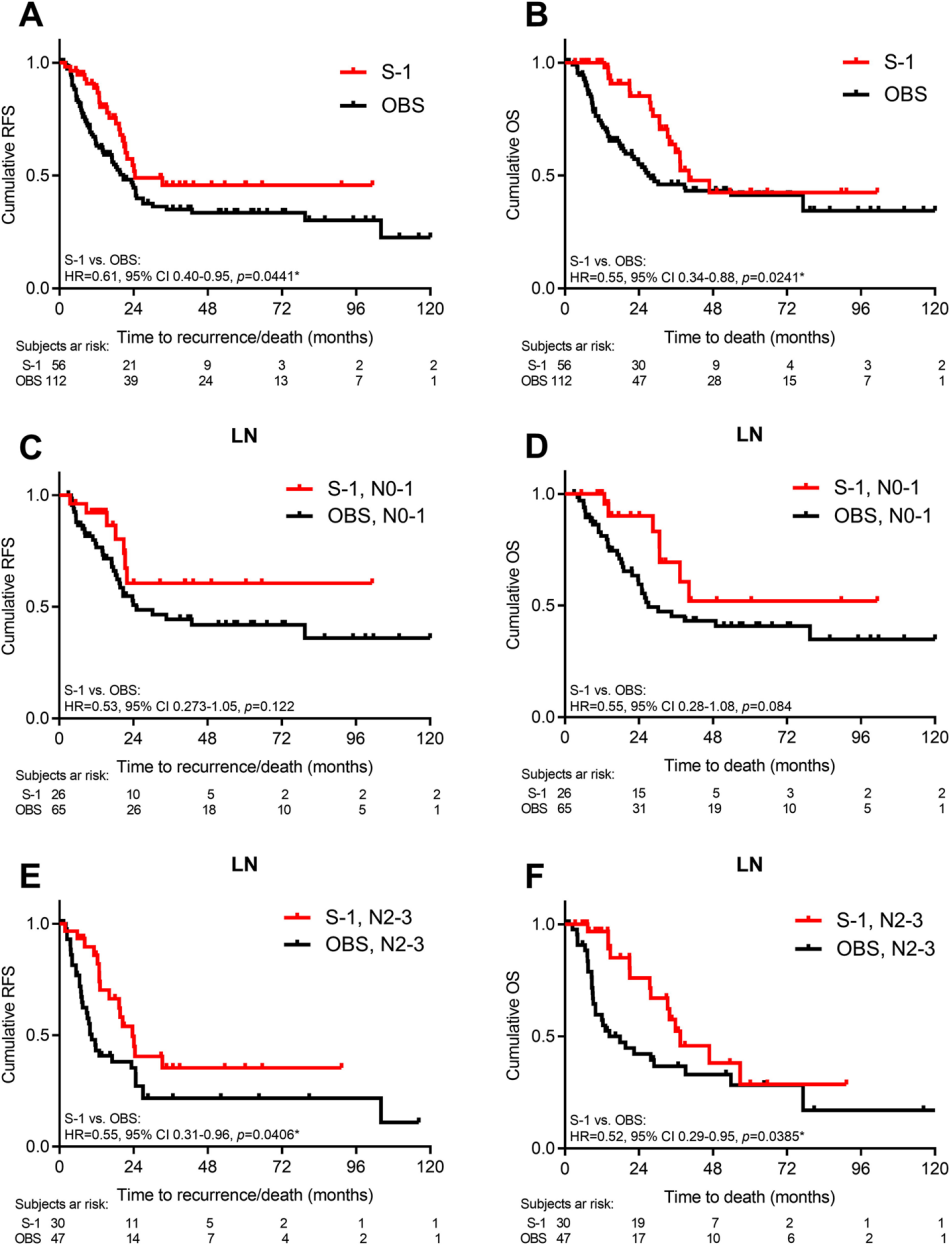
Survival: S-1 vs. close observation. RFS and OS by adjuvant S-1 or OBS (**A**-**B**), lymph node stage of N0-1 (**C**-**D**), and N2-3 (**E**-**F**). Log-rank test, *p*< 0.05 as statistically significant and shown as *

In analysis 2, Group S-1 was associated with favorable survival outcomes as compared with OBS (median RFS/OS: S-1 vs. OBS, 24.4/40.2 vs. 20.7/27.0 months, HR = 0.62/0.55, *p* = 0.041/0.024; Fig. 3A and B). The survival differences were less prominent in patients with N0–1 disease (5-yr RFS/OS rates: S-1 vs. OBS, 61.0%/52.0% vs. 42.0%/40.7%, HR = 0.68/0.67, *p* = 0.122/0.084; Fig. 3C and D). Patients with N2–3 disease had a better survival when treated with S-1 as compared with OBS (median RFS/OS: S-1 vs. OBS, 23.6/37.4 vs. 10.4/17.8 months, HR = 0.55/0.52, *p* = 0.041/0.039; Fig. 3E and F). However, patients who received S-1 had significant survival benefits in stage IB-II but less evident in stage III disease, respectively (Stage IB-II in 5-yr RFS/OS rates: S-1 vs. OBS, 72.2%/72.5% vs. 42.3%/50.4%, HR = 0.29/0.26, *p* = 0.013/0.018; Supplementary 3A and 3B) (Stage III in median RFS/OS: S-1 vs. OBS, 20.4/35.8 vs. 12.0/21.8 months, HR = 0.71/0.63, *p* = 0.214/0.130; Supplementary 3C and 3D).

### Adverse events and dosage intensity

We present the adverse events and the grading in Table 3 for the total number of evaluable patients (S-1 monotherapy, *n* = 61; platinum-based doublets, *n* = 105). There were no deaths attributable to the adjuvant chemotherapies. Anemia was the most common adverse event in all patients and was significantly higher in the platinum-based doublets as compared with S-1 (grade III/IV anemia: S-1 vs. combination therapy, 4.9% vs. 8.6%, *p* = 0.026). Grade III/IV thrombocytopenia, neutropenia and renal insufficiency were prevalent in the platinum-based doublets (thrombocytopenia, 15.2%; neutropenia, 4.8%; renal insufficiency, 3.8%). Scattered cases of severe diarrhea, anorexia, mucositis, and palmar plantar erythrodysesthesia were also observed in patients who received platinum-based doublets. In the S-1 group, except for a few cases with grade III/IV hepatitis (*n* = 2), thrombocytopenia (*n* = 1), and neutropenia (n = 1), there were no additional warning toxicities reported. One patient developed S-1-related grade III drug eruptions that were relieved after discontinuation and supportive care. The average dose intensity of S-1 was 40.9 ± 13.6 mg in patients with a body surface area (BSA) < 1.25 m^2^, 47.4 ± 13.4 mg with a BSA from 1.25 to 1.50 m^2^, and 58.5 ± 12.4 mg with a BSA > 1.50 m^2^ per day, which were lower than the suggested dose from the published studies, with an average relative intensity of 73.6% (Supplementary 4). 10.9% of the patients required a dose reduction due to intolerable toxicity. The median duration of S-1 was 8.3 months, respectively.

**Table 3:** Adverse events, according to the treatment

	S-1 (n = 61)				Platinum-based doublets (n = 105)^**a**^				
Events	Total, n	Grade I/II, n	Grade III/IV, n	Grade III/IV, (%)	Total, n	Grade I/II, n	Grade III/IV, n	Grade III/IV, (%)	***p***^**b**^
**Any events**	90	82	8	**–**	249	204	45	**–**	–
**Thrombocytopenia**	7	6	1	**1.6**	41	25	16	**15.2**	3.2e-4*
**Anemia**	22	19	3	**4.9**	58	49	9	**8.6**	0.026*
**Neutropenia**	3	2	1	**1.6**	22	17	5	**4.8**	0.010*
**Renal insufficiency**	5	5	0	**0**	19	15	4	**3.8**	0.129
**Hepatitis**	13	11	2	**3.3**	15	13	2	**1.9**	0.342
**Anorexia**	22	22	0	**0**	37	33	4	**3.8**	0.952
**Diarrhea**	9	9	0	**0**	10	9	1	**1.0**	0.443
**Vomiting**	6	6	0	**0**	31	30	1	**1.0**	3.5e-3*
**Mucositis**	1	1	0	**0**	5	4	1	**1.0**	0.416
**PPE**	0	0	0	**0**	9	8	1	**1.0**	–
**Skin eruptions**	2	1	1	**1.6**	2	1	1	**1.0**	0.625

a. PFL (*n* = 58), XELOX (*n* = 41) and FOLFOX (n = 6)

b. Comparisons with incidence of all events

PPE, palmar plantar erythrodysesthesia

### Subgroup analysis

**Fig. 4:**
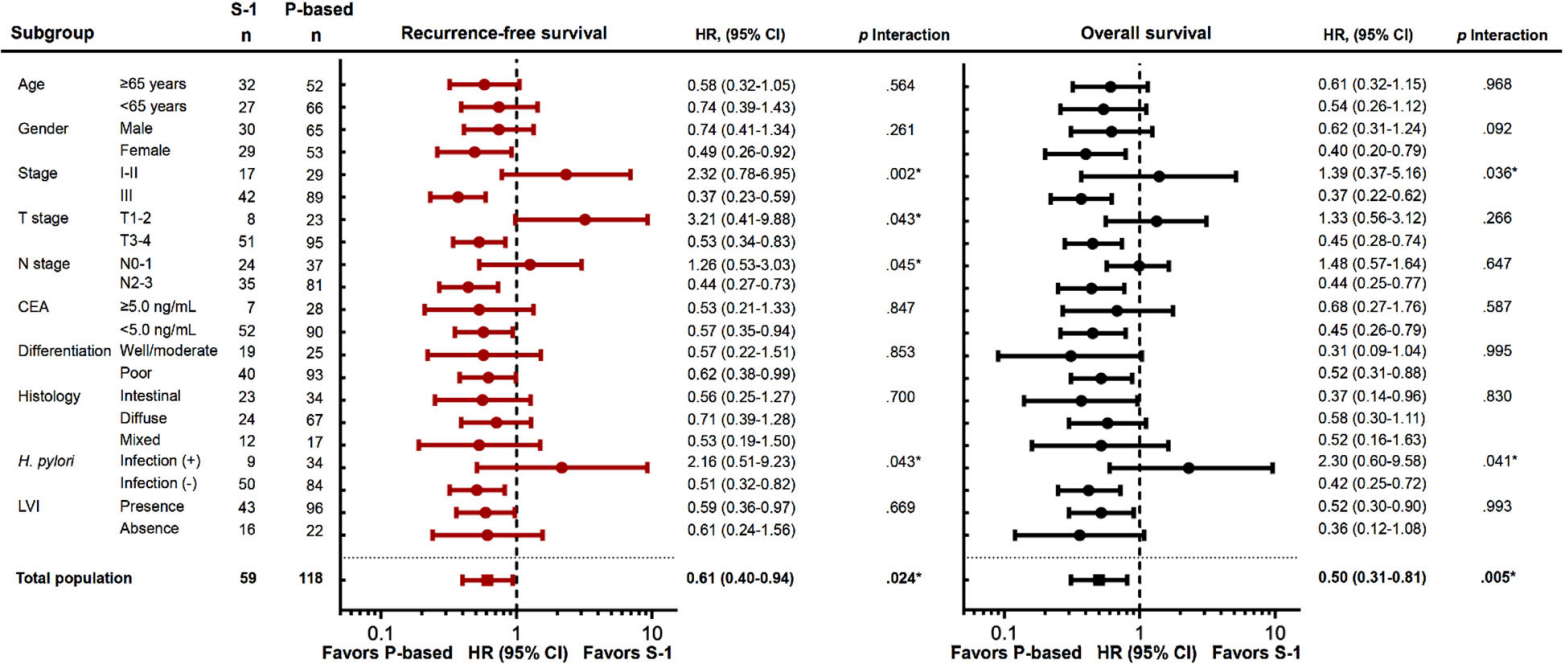
Subgroup analysis. *p for interaction* < 0.05 as statistically significant and shown as *

The subgroup analyses for RFS and OS are shown in Fig. 4 using forest plotting. In the total population, adjuvant platinum-based doublets were associated with favorable outcomes as compared with S-1 in the PPS-matched high-risk patients. In general, most subgroups favored platinum-based doublets. AJCC stage, T stage, N stage and *H. pylori* infection showed interactions between adjuvant therapies and these factors in RFS, and AJCC stage and *H. pylori* infection in OS, respectively.

### Factors associated with adjuvant S-1

We evaluated the factors associated with selecting adjuvant S-1 versus platinum-based doublets using a binary logistic regression, and the results are shown in Table 4. We observed that age, N stage, serum CEA, AJCC stage and tumor differentiation were associated factors in the univariate regression. Following multivariate adjustments, increased age, N0–2, low serum CEA and stage I-II disease were significant factors for adjuvant S-1 rather than platinum-based doublets.

**Table 4:** Associated factors for the selection of adjuvant S-1

		Univariate regression			Multivariate regression		
	n	OR	95% CI	***p***	OR	95% CI	***p***
**Age, increased by 1 year**	**212**	**1.04**	**1.02–1.08**	4.1e-4*	**1.05**	**1.03–1.09**	1.3e-4*
**N stage**							
**N3**	**87**	**1.00**					
**N1–2**	**103**	**3.12**	**1.22–8.47**	0.020*	**3.42**	**1.05–10.01**	0.019*
**N0**	**22**	**7.77**	**2.86–22.57**	8.0e-5*	**7.80**	**2.62–24.95**	3.2e-4*
**CEA**							
**≥ 5 ng/mL**	**54**	**1.00**					
**< 5 ng/mL**	**118**	**4.11**	**1.85–10.48**	0.001*	**3.85**	**1.63–10.27**	0.004*
**AJCC stage**							
**III**	**59**	**1.00**					
**I-II**	**153**	**3.60**	**1.92–6.83**	7.4e-4*	**2.42**	**1.01–5.81**	0.047*
Tumor differentiation							
Poor	162	1.00					
Well/Moderate	50	1.82	0.94–3.52	0.073	0.90	1.41–1.94	0.791
LVI							
Presence	164	1.00					
Absence	48	1.79	0.91–3.45	0.090			
Lauren classification							
Intestinal	67	1.00					
Diffuse	108	1.70	0.88–3.29	0.113			
Mixed	37	0.98	0.43–2.27	0.958			
*H. pylori* infection							
Presence	52	1.00					
Absence	160	1.70	0.84–3.64	0.150			
Gender							
Female	95	1.00					
Male	117	0.77	1.43–1.38	0.377			
Borrmann classification							
Non-infiltrative	47	1.00					
Infiltrative	165	1.30	0.65–2.56	0.446			
ECOG							
< 2	197	1.00					
≥ 2	15	1.29	0.42–481	0.670			
HER2 status							
Positive	14	1.00					
Negative	198	1.17	0.37–4.38	0.801			
T stage							
T3–4	170	1.00					
T1–2	42	1.04	0.51–2.22	0.919			

OR, odds ratio

## Discussion

Adjuvant S-1 has extended the therapeutic options in stage II to III gastric cancer patients who have received D2 gastrectomy, notably in Asia [18]. The present study provides additional information on S-1 versus close observation and conventional platinum-based chemotherapies based on real-world practice. Our results indicated that patients with a high risk of recurrence had an intermediate survival between close observation and platinum-based doublets when treated with adjuvant S-1, and had fewer adverse reactions and better tolerability as compared with platinum-based doublets. In an era prompting aggressive adjuvant therapies for high-risk diseases, curative gastrectomy alone is no longer being considered as a standard of care. Sakuramoto et al. proposed that adjuvant S-1 is superior to D2 gastrectomy alone in stage II to III gastric cancer in terms of both RFS and OS [14, 21]. In the CLASSIC study, adjuvant capecitabine/oxaliplatin (XELOX) significantly improved survival outcomes at the cost of more adverse events [22]. In patients with a particularly high risk of recurrence, the role of more aggressive therapies has been intensively investigated. The ARTIST-2 trial evaluated the role of adjuvant S-1/oxaliplatin (SOX), SOX plus CCRT, and S-1 monotherapy in LN-positive patients, where it was concluded that S-1-based doublets, irrespective of whether the patient underwent radiotherapy or not, were superior to S-1 in terms of disease-free survival and met the prespecified endpoint early [23]. Yoshida et al. also reported the therapeutic efficacy of docetaxel/S-1 in stage III, ≥T2 or LN-positive patients over S-1, with a significantly higher incidence of grade III to IV treatment-related toxicities [15]. Other combinations, such as oxaliplatin or cisplatin plus S-1, have been considered to be feasible options but were found to be relatively more toxic than S-1 monotherapy [11, 24–26]. The present study reveals compatible results to these clinical trials that doublets are superior than monotherapy in high-risk, such as T3–4 or LN-positive, patients. Also, our results suggest that adjuvant S-1 is well-tolerated and comparable in efficacy to other combinatorial regimens in patients with selected risk of recurrence. Although intensive protocols are promising, selecting an optimal adjuvant chemotherapy remains a difficult task that requires a balance between the therapeutic benefits and toxicity.

Postoperative lymph node status plays an essential role in prediction of recurrence or progression of the disease. Our results indicated that S-1 correlated with unfavorable survivals in N2–3 but comparable in N0–1 disease as compared with platinum-based doublets. In the ACTS-GC study, the therapeutic efficacy of S-1 monotherapy was less evident in patients with stage III, T3–4 or N2–3 disease [21]. A retrospective analysis indicated that patients with N3 disease or a lymph node ratio (LNR) > 0.25 did better with adjuvant XELOX rather than with S-1 [27]. Hsieh et al. also suggested that stage III or LNR > 0.21 are significant prognostic factors in patients receiving S-1 versus non-S-1 doublet chemotherapies [28]. Similarly, in the JACCRO-GC-07 trial, despite the fact that docetaxel/S-1 was superior to S-1 monotherapy in the primary analysis, the benefit disappeared in patients with N0–1 disease [15]. In addition, we noticed that patients with *H. pylori* infection had a trend of survival benefits when treated with S-1 as compared with platinum-based doublets. *H. pylori* played an unclear role as a prognostic factor in patients with gastric cancer [29, 30]. Nishizuka et al. reported that *H. pylori*-infected patients had superior survivals when treated with S-1, which was compatible with our results [31]. Host antitumor immunity may be enhanced by *H. pylori* infection and fluoropyrimidine-mediated eradication of myeloid-derived suppressor cells, which may contribute to a survival advantage [32]. Together our results may provide information on the role of S-1 in patients with selected risk of recurrence or specific characteristics as adjuvant therapy.

Good tolerance and limited warning toxicities with S-1 was observed in the present study. We found only a few cases of grade III to IV events, such as anemia, hepatitis, and skin eruptions in contrast to a higher risk of anemia, thrombocytopenia, neutropenia, and renal insufficiency with the use of platinum-based doublets. In addition, a low demand of dose reduction (10.9%) was noticed, despite the fact that the patients were potentially under-dosed (73.6% as compared with reference trials) in a real-world practice environment. Kim et al. reported a dose of S-1 at 40 mg/m^2^ twice daily in D1–28 every 6 weeks for one year and concluded that 46.3% of the patients required a dose modification, and 16.1% discontinued treatment due to persistent toxicity [33]. Yamatsuji et al. compared a 4-week administration period followed by two weeks of rest (4-w/2-w) with a two week administration period followed by one week of rest (2-w/1-w) and revealed that the latter had a higher completion rate, higher relative dose intensity, and similar toxicities [34]. In addition, the optimal dosage of S-1 is potentially confounded by ethnicity. Yang et al. revealed that S-1 at a dose of 35 mg/m^2^ twice daily was better tolerated in Asian patients in combination with oxaliplatin [35]. However, another pharmacokinetic study revealed a poor tolerance in a Western population, and the dose was reduced to 25 mg/m^2^ twice daily [17]. The discrepancy is partially derived from the pharmacogenetic polymorphisms of cytochrome P 2A6 (CYP2A6), which contribute to a distinctive S-1 elimination efficiency [36]. Further studies are required to determine the patient-tailored optimal dosage of S-1, both in mono- or combinatorial therapies.

We found that physicians preferred S-1 as adjuvant chemotherapy in patients with increased age, N0–2, low serum CEA and stage I-II disease. Lee et al. suggested that S-1 was more likely to be selected as adjuvant chemotherapy for patients over 65 years of age, whereas XELOX was selected for patients with T4, N2–3 and stage III disease, corresponding to our results [37]. Terashima et al. evaluated the tumor gene expression profiles of 102 patients who received adjuvant chemotherapies via a DNA microarray and found that the cluster of differentially expressed genes that favored S-1 was enriched with immune-related genes [38]. Together, these results highlight physician preferences and potential selection biomarkers for those who may derive benefits from S-1 containing adjuvant therapies. Recently, perioperative chemotherapies have gained awareness for the efficacy on locally-advanced or high-risk patients with resectable or borderline resectable gastric cancer. Various combinations of fluoropyrimidines, platinums, anthracyclines, or taxanes have been adopted to improve the resectability, surgical outcome, and long-term survival, as well as to reduce recurrence [4–6, 22, 39, 40]. The addition of S-1 to these agents is being intensively investigated [41–43]. Furthermore, the results are promising in the combination of S-1, nivolumab, oxaliplatin, and capecitabine in advanced or recurrent gastric cancer [44]. More clinical studies are required to delineate the role of S-1, both in the frontline or adjuvant setting and in the treatment of gastric cancer.

The strength of the present study is the real-world information of S-1 outside of clinical trial settings, which is more closely related to actual clinical scenarios. In addition, we incorporated PPS matching to exclude baseline heterogeneity and enhance between-group comparability. Third, we provided the dosages and adverse reactions for S-1 in comparison with other combinatorial regimens. However, there are some limitations to this study. It is a retrospective analysis that cannot address prospective consequential imputations. Also, we included patients who received S-1 monotherapy in the absence of S-1-based combinations, which have gradually become the standard of care in high-risk populations. In addition, despite the efforts we made to control for differences of patients via PPS matching, we still were unable to eradicate possible exclusion bias and undetermined imbalances. Lastly, the patients were collected from a single institution, and the case number was not significantly large enough to have an excellent statistical power and the results might be affected by events or deaths out of random effects. Overall, the study still provided information concerning adjuvant S-1 in the treatment of resectable gastric cancer and a comparison with close observation and platinum-based doublets.

## Conclusion

In the present study, patients with D2-resected locally-advanced gastric cancer who received adjuvant S-1 had an intermediate survival between close observation and platinum-based doublets and had fewer adverse reactions as compared with platinum-based doublets. S-1 was relatively under-dosed and found to be selected by physicians based on age, LN status, serum CEA and stage. More prospective studies are required to delineate the optimal selection of patients who may benefit from S-1 or S-1-based combinations in gastric cancer.

## Supplementary Information

**Additional file 1.** Supplementary 1: Subsequent therapies for the study population. Supplementary 2: Survival of S-1 vs. platinum-based doublets. Supplementary 3: Survival of S-1 vs. close observation. Supplementary 4: Dose intensity, reduction, and duration of S-1.s

## Data Availability

The data that support the findings of this study are available from Cancer Registry of the Electronic Medical Records in National Cheng Kung University Hospital but restrictions apply to the availability of these data, which were used under specific permission for the current study, and so are not publicly available. Data are however available from the authors upon reasonable request and with permission of Clinical Research Center, National Cheng Kung University Hospital, Tainan, Taiwan. em74820@mail.hosp.ncku.edu.tw. No. 138, Sheng-Li Road, Tainan 70403, Taiwan. Tel: + 886–6–235-3535 ext. 4817.

## Abbreviations

HER2: human epidermal growth factor receptor-2
CEA: carcinoembryonic antigen
HSD: honestly significant difference
S-1: tegafur/gimeracil/octeracil
OBS: observation
RFS: recurrence-free survival
OS: overall survival
CCRT: concurrent chemotherapy
GEJ: gastroesophageal junction
AJCC: American Joint Committee on Cancer
*H. pylori*: Helicobacter pylori
CTCAE: Common Toxicity Criteria of the National Cancer Institute
ANOVA: analysis of variance
PPS: propensity scores
LN: lymph node
CA19–9: cancer antigen 19–9
LVI: lymphovascular invasion
PFL: cisplatin/fluorouracil/leucovorin
LNR: lymph node ratio
CYP: cytochrome P
