# Supplementary material for: Surgery alone, adjuvant tegafur/gimeracil/octeracil (S-1), or platinum-based chemotherapies for resectable gastric cancer: real-world experience and a propensity score matching analysis
Source: BMC Cancer. 2021 Jul 9;21:796. doi: 10.1186/s12885-021-08487-z (PMC8268293; doi:10.1186/s12885-021-08487-z)
Supplement: Supplementary file 1 — Additional file 1. Supplementary 1: Subsequent therapies for the study population. Supplementary 2: Survival of S-1 vs. platinum-based doublets. Supplementary 3: Survival of S-1 vs. close observation. Supplementary 4: Dose intensity, reduction, and duration of S-1.s [file 12885_2021_8487_MOESM1_ESM.pdf]

**Title Page**

**Original Article**

# Surgery Alone, Adjuvant Tegafur/gimeracil/octeracil (S-1), or Platinum-based Chemotherapies For Resectable Gastric Cancer: Real-world Experience and A Propensity Score Matching Analysis


Chih-Chieh Yen[1,2], Yan-Shen Shan[2,3], Ying-Jui Chao[3], Ting-Kai Liao[4], I-Shu Chen[5], Hsuan-Yi Huang[6], I-Ting Liu[7] and Chia-Jui Yen[7]

[1]Division of Hematology/ Oncology, Department of Internal Medicine, National Cheng Kung University Hospital Douliou Branch, Yunlin, Taiwan

[2]Institute of Clinical Medicine, School of Medicine, National Cheng Kung University, Tainan, Taiwan

[3]Department of Surgery, National Cheng Kung University Hospital, College of Medicine, National Cheng Kung University, Tainan, Taiwan

[4]Department of Surgery, National Cheng Kung University Hospital Douliou Branch, YunLin, Taiwan

[5]Department of Surgery, Kaohsiung Veterans General Hospital, Kaohsiung, Taiwan

[6]Division of Colorectal Surgery, Department of Surgery, Chi Mei Medical Center, Tainan, Taiwan

[7]Department of Oncology, National Cheng Kung University Hospital, College of Medicine, National Cheng Kung University, Tainan, Taiwan

Corresponding Author:

Chia-Jui Yen, MD., PhD.
Department of Oncology, National Cheng Kung University Hospital, College of Medicine, National Cheng Kung University, Tainan, Taiwan
yencj@mail.ncku.edu.tw
No. 138, Sheng-Li Road, Tainan 70403, Taiwan
Tel: +886-6-235-3535 ext. 4620

# Supplementary Materials

## Contents

Supplementary 1: Subsequent therapies for the study population ··········3

Supplementary 2: Survival of S-1 *vs.* platinum-based doublets··········4

Supplementary 3: Survival of S-1 *vs.* close observation··········5

Supplementary 4: Dose intensity, reduction, and duration of S-1··········6

## Supplementary 1: Subsequent therapies for the study population

| | **Analysis 1: S-1 vs. P** | | | | **Analysis 2: S-1 vs. OBS** | | | |
|---|---|---|---|---|---|---|---|---|
| | 2nd line therapies, n (%)[a] | | 3rd line therapies, n (%)[b] | | 2nd line therapies, n (%)[a] | | 3rd line therapies, n (%)[b] | |
| | S-1, n=30 | P, n=71 | S-1, n=13 | P, n=26 | S-1, n=26 | OBS, n=46 | S-1, n=9 | OBS, n=13 |
| **XELOX/FOLFOX** | 12 (40) | 15 (21) | 0 | 1 (4) | 10 (38) | 10 (22) | 0 | 0 |
| **5-FU/S-1** | 4 (13) | 9 (13) | 2 (15) | 3 (12) | 3 (12) | 8 (17) | 2 (22) | 2 (15) |
| **Taxanes** | 1 (3) | 10 (14) | 1 (8) | 2 (8) | 2 (8) | 3 (7) | 1 (11) | 2 (15) |
| **PFL** | 2 (7) | 4 (6) | 0 | 1 (4) | 0 | 7 (15) | 0 | 0 |
| **Taxanes + RAM** | 1 (3) | 4 (6) | 2 (15) | 4 (15) | 1 (4) | 2 (4) | 1 (11) | 2 (15) |
| **RT** | 1 (3) | 1 (1) | 0 | 3 (12) | 1 (4) | 1 (2) | 0 | 0 |
| **ICPi** | 1 (3) | 2 (3) | 2 (15) | 4 (15) | 1 (4) | 1 (2) | 0 | 1 (8) |
| **Re-operation** | 0 | 2 (3) | 0 | 0 | 2 (8) | 0 | 0 | 0 |
| **Others** | 0 | 4[c] (6) | 0 | 1[d] (4) | 0 | 1[d] (2) | 0 | 0 |
| **RAM** | 0 | 0 | 2 (15) | 2 (8) | 0 | 0 | 1 (11) | 0 |
| **BSC** | 8 (27) | 20 (28) | 4 (31) | 5 (19) | 6 (23) | 13 (28) | 4 (44) | 6 (46) |

a. 2nd line therapies were referred as any subsequent treatments post surgery ± adjuvant chemotherapies. Case numbers were defined by patients who were eligible for the subsequent therapies listed.
b. 3nd line therapies were referred as any subsequent treatments post 2nd line therapies.
c. XELOX + trastuzumab (n=2), ECF (n=1) and lapatinib (n=1).
d. Irinotecan (n=1).

XELOX, capecitabine/oxaliplatin; FOLFOX, 5-FU/leucovorin/oxaliplatin; 5-FU, fluoropyrimidines; PFL, cisplatin/5-FU/leucovorin; RAM, ramucirumab; RT, radiotherapy; ICPi, immune checkpoint inhibitors; BSC, best supportive care; ECF, epirubicin/cisplatin/5-FU.

## Supplementary 2: Survival: S-1 *vs.* platinum-based doublets

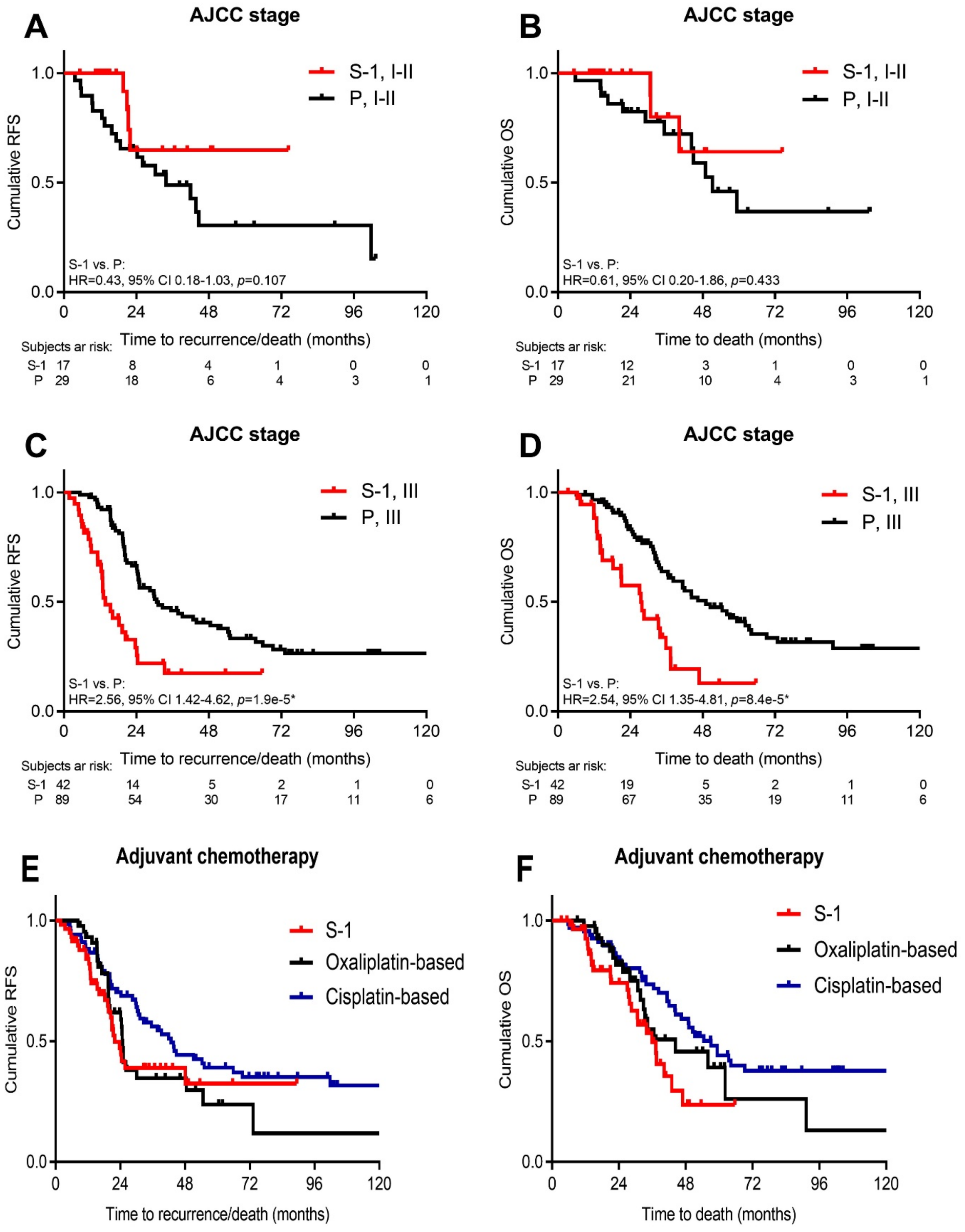


RFS and OS by AJCC pathological stage IB-IIIA (A-B), stage IIIB/IIIC (C-D), and chemotherapy regimens (E-F).

Log-rank test, $p < 0.05$ as statistically significant and shown as *.

**Supplementary 3: Survival: S-1 *vs.* close observation**

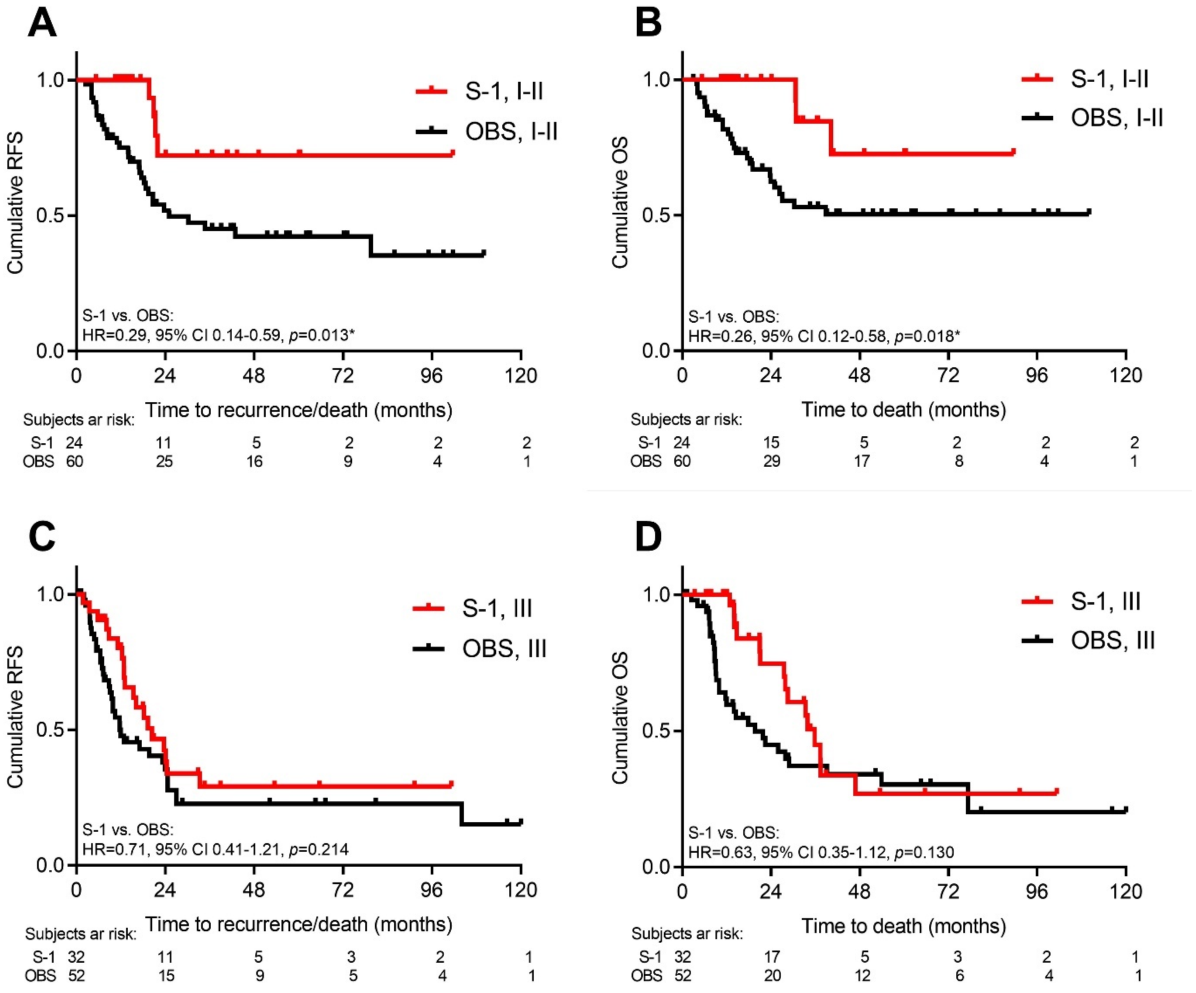


RFS and OS by AJCC pathological stage IB-IIIA (A-B) and stage IIIB/IIIC (C-D).

Log-rank test, *p*< 0.05 as statistically significant and shown in *.

## Supplementary 4: Dose intensity, reduction, and duration of S-1

| | Recommended dose[a] | | The present study (n=64) | | | |
|---|---|---|---|---|---|---|
| **S-1 dose** | Dose, mg/day | Average dose intensity[b], mg/day | Average dose intensity, mg/day ± SD | Relative dose intensity, % | Requiring a dose reduction, % | Median duration, months |
| **BSA <1.25 $m^2$** | 80 | 53.3 | 40.9 ±13.6 | 76.7 | 9.1 | 6.4 |
| **1.25-1.50 $m^2$** | 100 | 66.7 | 47.4 ±13.4 | 71.1 | 8.7 | 8.8 |
| **>1.50 $m^2$** | 120 | 80.0 | 58.5 ±12.4 | 73.1 | 12.5 | 10.1 |

a. Recommended dose is followed by ACTS-GC[14] and JACCRO-GC-07[15] studies and are calculated as the reference dose of 100%.

b. Average dose is determined by the accumulated dose each cycle divided by cycle days (including treatment-free days) as daily dose intensity. A consecutive 28 days every 42 days in the ACTS-GC and 14 days every 21 days in the JACCRO-GC-07 studies are calculated as one cycle.

BSA, body surface area; SD, standard deviation
